# John’s Equation-based Consistency Condition and Corrupted Projection Restoration in Circular Trajectory Cone Beam CT

**DOI:** 10.1038/s41598-017-05249-5

**Published:** 2017-07-07

**Authors:** Jianhui Ma, Shuyu Wu, Hongliang Qi, Bin Li, Hao Yan, Linghong Zhou, Yuan Xu

**Affiliations:** 1 School of Biomedical Engineering, Southern Medical University, Guangzhou, 510515 China; 2 Cyber Medical Corporation, Xi’an, 710000 China

## Abstract

In transmitted X-ray tomography imaging, the acquired projections may be corrupted for various reasons, such as defective detector cells and beam-stop array scatter correction problems. In this study, we derive a consistency condition for cone-beam projections and propose a method to restore lost data in corrupted projections. In particular, the relationship of the geometry parameters in circular trajectory cone-beam computed tomography (CBCT) is utilized to convert an ultra-hyperbolic partial differential equation (PDE) into a second-order PDE. The second-order PDE is then transformed into a first-order ordinary differential equation in the frequency domain. The left side of the equation for the newly derived consistency condition is the projection derivative of the current and adjacent views, whereas the right side is the projection derivative of the geometry parameters. A projection restoration method is established based on the newly derived equation to restore corrupted data in projections in circular trajectory CBCT. The proposed method is tested in beam-stop array scatter correction, metal artifact reduction, and abnormal pixel correction cases to evaluate the performance of the consistency condition and corrupted projection restoration method. Qualitative and quantitative results demonstrate that the present method has considerable potential in restoring lost data in corrupted projections.

## Introduction

In cone-beam computed tomography (CBCT), the projections acquired are frequently corrupted because of the limitations of physical hardware or data processing. For example, corrupted detector cells can induce the corresponding pixels in the projection to behave abnormally, and consequently, result in ring artifacts superimposed on the reconstructed image. These artifacts can seriously affect the extraction of diagnostic information from the reconstructed images^1, 2^; thus, an abnormal pixel correction method is essential to eliminate ring artifacts^3, 4^. A typical correction method is the sinogram-based correction method, in which the abnormal pixels are initially detected using a wavelet-based technique before the abnormal pixels are estimated via linear interpolation^5^. However, linear interpolation is weak in a high-frequency domain; thus, a better restoration method is necessary to improve the quality of reconstructed images.

Data inaccuracy is also a common problem in artifact correction. For example, scattered photons severely degrade image quality^6–8^. To address the issue of scatter correction, various approaches have been proposed, such as anti-scatter grid usage^9^, bow-tie filter compensation^10^, Monte Carlo simulation^11, 12^, analytical computation^13^, and scatter kernel calculation^14^. Another well-known type of scatter correction methods is scatter measurement, whose concept is to accurately measure scatter signals with the aid of additional hardware^15, 16^. A typical scatter-measurement method is measuring the scattering intensity with a beam-stop array (BSA) in the extra scan to obtain the influence of scatter via 2D spatial interpolation based on the measured data^17^. Zhu^18^ also proposed a moving BSA method, which integrated extra scan and normal scan to reduce the total dose significantly. The primary photon beam in each view is not blocked at a fixed position because of the movement of BSA; however, the projection remains corrupted in each view. Thus, restoring lost data in each projection is a vital step in the BSA scatter correction workflow. Conventional spatial interpolation can only be utilized among a single projection because the projections are scanned in a circular trajectory, thereby limiting the restoration of high-frequency components and producing streak artifacts^16^.

Besides, metal objects such as metal implants, dental fillings, and prostheses possess high densities, therefore the presence of high-density objects in field of view (FOV) can lead to the severe metal artifacts which seriously degrade image diagnosis value^19–21^. Generally, noise, exponential edge gradient effect, beam hardening artifacts, and scatter artifacts are all the causes of metal artifacts^19^. The aim of metal artifact reduction methods is to restore the corrupted data caused by metal objects, therefore an efficient method is required here to improve the image quality degraded by above described artifacts.

CBCT projections are typically redundant. Therefore, we apply consistency conditions to restore lost data in corrupted projections^22, 23^. Consistency conditions refer to the constraints between the current projection and its adjacent projections. Their specific forms are hyperbolic equations, which are linear constant coefficient partial differential equation (PDE) and homogeneous second-order PDE. Fritz John first proposed an ultra-hyperbolic PDE, which later became known as John’s equation, to solve line integral problems^24^. Computed tomography (CT) reconstruction is based on radon transform theory; thus, the projection data naturally satisfy ultra-hyperbolic PDEs^25, 26^. However, John’s equation is difficult to utilize because of the implementation of its second-order PDE. To address this issue, Patch^27^ converted the second-order PDE into a family of first-order ordinary differential equations (ODEs) in the frequency domain. On the basis of her deduced ODEs, corrupted or unmeasured projections can be acquired using the information of the adjacent projections in helical CT. However, the equation derived by Patch in the frequency domain requires a derivative with respect to pitch *z*; thus, Patch’s equation is difficult to apply to helical CT data^28^. Moreover, pitch *z* is constant in circular trajectory CBCT; thus, the derivative with respect to pitch *z* is meaningless. Therefore, Patch’s equation cannot be utilized for CBCT data in a circular trajectory.

We consider these issues to derive a new consistency condition from John’s equation and propose a method to restore lost data in corrupted projections in circular trajectory CBCT. In particular, the relationship of the geometry parameters in circular trajectory CBCT is utilized to convert an ultra-hyperbolic PDE into a first-order ODE in the frequency domain. A corresponding method is established with the newly derived equation to restore lost data in corrupted projections in circular trajectory CBCT. The method is tested in scatter correction, metal artifact reduction, and abnormal pixel correction cases. Qualitative and quantitative results show that the proposed method is promising in restoring corrupted projections in circular trajectory CBCT.

The remainder of this paper is organized as follows. Section 2 introduces John’s equation and the geometry configuration of circular trajectory CBCT. Then, it describes the equation derivations and the projection data restoration method. Sections 3 and 4 respectively report in detail the experiments and the results of the proposed method in the scatter correction, abnormal pixel correction, and metal artifact reduction cases. Finally, the discussion of the results and the conclusions drawn from this study are presented in Sections 5 and 6, respectively.

## Method

### John’s equation

In 1938, Fritz John derived the ultra-hyperbolic PDE, which can solve the problem of line integrals on a characteristic surface. Let *ε* and *η* denote the X-ray source and detector cell, respectively. John’s equation^24^ can be expressed as follows:

1 $$(\frac{{\partial }^{2}}{\partial {\eta }_{i}\partial {\varepsilon }_{j}}-\frac{{\partial }^{2}}{\partial {\eta }_{j}\partial {\varepsilon }_{i}})u(\varepsilon ;\eta )=0,\,i,j=1,2,3$$

where *u* denotes a set of line integrals of the object function *f*, which represents a sufficiently differentiable function of an object along the line through *ε* and *η*, and can be expressed as

2 $$u(\varepsilon ;\eta )={\int }_{R}f(\varepsilon +t(\eta -\varepsilon ))dt$$

### Geometry configuration of circular trajectory CBCT

**Figure 1:**
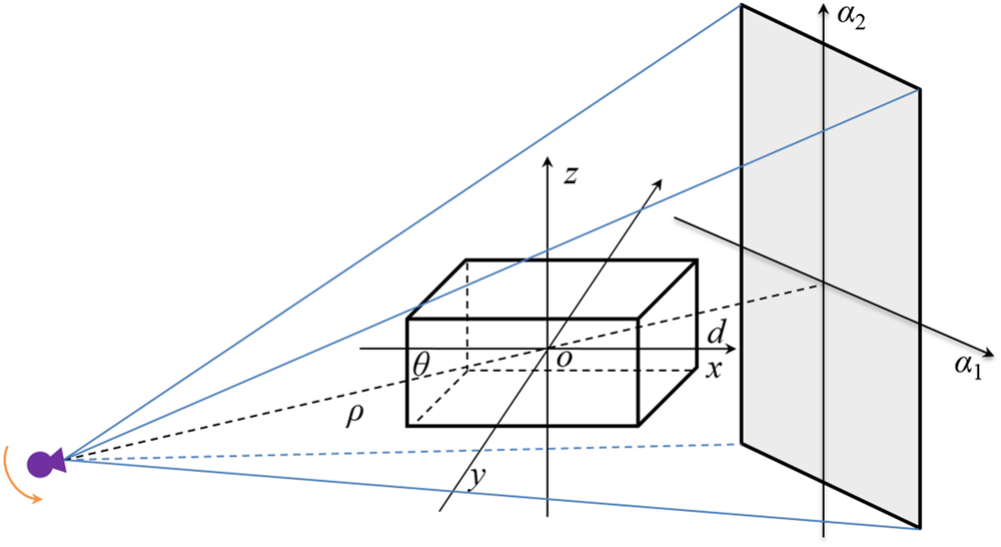
Geometry configuration of circular trajectory CBCT.

In this section, we introduce circular trajectory CBCT geometry, which is utilized to convert the ultra-hyperbolic PDE into an ODE. As shown in Fig. 1, *o-xyz* is a global coordinate system, where *o* is the rotation isocenter; (*α*_1_, *α*_2_) is the local coordinate system of the flat panel detector, where *α*_2_ is parallel to the *z*-axis and *α*_1_ is orthogonal to *α*_2_. The rotation angle of the X-ray source and detector around the *x*-axis is *θ*; *ρ* and *d* denote source-to-isocenter distance and isocenter-to-detector distance, respectively.

### Consistency condition in circular trajectory CBCT

In this section, we would like to explain further why Patch’s consistency condition method cannot be directly utilized in circular trajectory CBCT. To calculate the unmeasured data in the projections acquired via helical CBCT geometry, Patch^27^ initially used geometry parameters of a third-generation helical CT system to rewrite the variables in John’s equation as follows:

3 $$\frac{{\partial }^{2}u}{\partial {\alpha }_{2}\partial \theta }-\rho \frac{{\partial }^{2}u}{\partial {\alpha }_{1}\partial z}=\frac{-1}{\rho +d}(2{\alpha }_{1}\frac{\partial u}{\partial {\alpha }_{2}}+{\alpha }_{1}{\alpha }_{2}\frac{{\partial }^{2}u}{\partial {\alpha }_{2}^{2}}+({(\rho +d)}^{2}+{\alpha }_{1}^{2})\frac{{\partial }^{2}u}{\partial {\alpha }_{1}\partial {\alpha }_{2}})$$

By transforming Eq. (3) into the frequency domain, Patch has converted John’s equation, which is a second-order PDE, into a first-order PDE as

4 $${k}_{2}{u}_{\theta }^{\ast }-\rho {k}_{1}{u}_{z}^{\ast }=\frac{i{k}_{2}}{\rho +d}(2{u}_{{k}_{1}}^{\ast }+{k}_{2}{u}_{{k}_{1}{k}_{2}}^{\ast }-{(\rho +d)}^{2}{k}_{1}{u}^{\ast }+{k}_{1}{u}_{{k}_{1}{k}_{1}}^{\ast })$$

where (*k*_1_, *k*_2_) is the corresponding frequency domain of (*α*_1_, *α*_2_), and the superscript * denotes a Fourier transform. The subscript denotes a derivative; for example, *u*_*θ*_ represents the derivative of *u* with respect to *θ*. Note that Eq. (4) is a first-order PDE for *θ* and *z*, and for fixed frequency component (*k*_1_, *k*_2_), it is referred to as a family of first-order ODEs.

Although Patch converts the complex John’s equation into an ODE, the helical coordinates in Eq. (4) consider *z* as a numerical variable. The change in variable *z* along the vertical axis destroys the homogeneity in a helical system^28^; thus, Patch’s equation is difficult to apply in practice. In theory, helical CT can degenerate into circular trajectory CBCT when *z* = 0. However, Eq. (4) contains a derivative of *z*; thus, this equation will not work when *z* is a constant. So, Eq. (4), which was deduced by Patch, could not be utilized in circular trajectory CBCT.

Therefore, we have derived a John’s equation-based consistency condition (JECC) and the derivation is described in detail in this section. To make John’s equation subject to the circular trajectory CBCT geometry, we firstly rewrite *ε* and *η* in terms of five parameters, namely, *α*_1_, *α*_2_, *ρ*, *d*, and *θ*. Then *ε* and *η* are defined by

5 $${\varepsilon }_{1}=\rho \,\cos \,\theta ,\,{\varepsilon }_{2}=\rho \,\sin \,\theta ,\,{\varepsilon }_{3}=0$$

6 $${\eta }_{1}=-{\alpha }_{1}\,\sin \,\theta -d\,\cos \,\theta ,{\eta }_{2}={\alpha }_{1}\,\cos \,\theta -d\,\sin \,\theta ,{\eta }_{3}={\alpha }_{2}$$

Eqs (5) and (6) can be transformed into the equivalent equations as follows:

7 $$\tan \,\theta =\frac{{\varepsilon }_{2}}{{\varepsilon }_{1}},\,\,{\alpha }_{1}=\frac{{\eta }_{2}{\varepsilon }_{1}-{\eta }_{1}{\varepsilon }_{2}}{\sqrt{{\varepsilon }_{1}^{2}+{\varepsilon }_{2}^{2}}},\,d=-(\frac{{\eta }_{1}{\varepsilon }_{1}+{\eta }_{2}{\varepsilon }_{2}}{\sqrt{{\varepsilon }_{1}^{2}+{\varepsilon }_{2}^{2}}}),\,{\rho }^{2}={\varepsilon }_{1}^{2}+{\varepsilon }_{2}^{2}$$

Combined Eqs (5), (6) with (7), the derivatives of the CBCT parameters with respect to *ε* and *η* are listed in Table 1.

**Table 1:** Derivatives of the CBCT parameters with respect to *ε* and *η*.

	*α*_1_	*α*_2_	*ρ*	*d*	*θ*
*ε*_1_	*−d·*sin*θ/ρ*	0	cos*θ*	*α*_1_ *·*sin*θ/ρ*	*−*sin*θ/ρ*
*ε*_2_	*d·*cos*θ/ρ*	0	sin*θ*	*−α*_1_ *·*cos*θ/ρ*	cos*θ/ρ*
*ε*_3_	0	0	0	0	0
*η*_1_	*−*sin*θ*	0	0	*−*cos*θ*	0
*η*_2_	cos*θ*	0	0	*−*sin*θ*	0
*η*_3_	0	1	0	0	0

For example, the result of *∂u*/*∂ε*_1_ is in the second cell of the second row, i.e., *∂α*_1_ *∂ε*_1_ = −*d*·sin*θ*/*ρ*.

According to the derivative of the compound function and the derivatives shown in Table 1, we can get

8 $$\begin{array}{rcl}\frac{\partial }{\partial {\varepsilon }_{1}} & = & \frac{\partial {\alpha }_{1}}{\partial {\varepsilon }_{1}}\cdot \frac{\partial }{\partial {\alpha }_{1}}+\frac{\partial {\alpha }_{2}}{\partial {\varepsilon }_{1}}\cdot \frac{\partial }{\partial {\alpha }_{2}}+\frac{\partial \rho }{\partial {\varepsilon }_{1}}\cdot \frac{\partial }{\partial \rho }+\frac{\partial d}{\partial {\varepsilon }_{1}}\cdot \frac{\partial }{\partial d}+\frac{\partial \theta }{\partial {\varepsilon }_{1}}\cdot \frac{\partial }{\partial \theta }\\  & = & \frac{\sin \,\theta }{\rho }(-d\frac{\partial }{\partial {\alpha }_{1}}+{\alpha }_{1}\frac{\partial }{\partial d}-\frac{\partial }{\partial \theta })+\,\cos \,\theta \frac{\partial }{\partial \rho }\end{array}$$

9 $$\frac{\partial }{\partial {\varepsilon }_{2}}=\frac{\cos \,\theta }{\rho }(d\frac{\partial }{\partial {\alpha }_{1}}-{\alpha }_{1}\frac{\partial }{\partial d}+\frac{\partial }{\partial \theta })+\,\sin \,\theta \frac{\partial }{\partial \rho }$$

10 $$\frac{\partial }{\partial {\varepsilon }_{3}}=0$$

11 $$\frac{\partial }{\partial {\eta }_{1}}=-(\sin \,\theta \frac{\partial }{\partial {\alpha }_{1}}+\,\cos \,\theta \frac{\partial }{\partial d})$$

12 $$\frac{\partial }{\partial {\eta }_{2}}=\,\cos \,\theta \frac{\partial }{\partial {\alpha }_{1}}-\,\sin \,\theta \frac{\partial }{\partial d}$$

13 $$\frac{\partial }{\partial {\eta }_{3}}=\frac{\partial }{\partial {\alpha }_{2}}$$

In order to facilitate writing, define the differential operator *L*

14 $$L=d\frac{\partial }{\partial {\alpha }_{1}}-{\alpha }_{1}\frac{\partial }{\partial d}+\frac{\partial }{\partial \theta }$$

Then John’s equation can be transformed as follows:

15 $$\{\begin{array}{c}(\frac{{\partial }^{2}}{\partial {\eta }_{1}\partial {\varepsilon }_{2}}-\frac{{\partial }^{2}}{\partial {\eta }_{2}\partial {\varepsilon }_{1}})u(\varepsilon ;\eta )=-(\frac{1}{\rho }\frac{\partial L}{\partial d}+\frac{{\partial }^{2}}{\partial {\alpha }_{1}\partial \rho })u({\alpha }_{1},{\alpha }_{2},\rho ,d,\theta )=0\\ (\frac{{\partial }^{2}}{\partial {\eta }_{1}\partial {\varepsilon }_{3}}-\frac{{\partial }^{2}}{\partial {\eta }_{3}\partial {\varepsilon }_{1}})u(\varepsilon ;\eta )=(\frac{\sin \,\theta }{\rho }\frac{\partial L}{\partial {\alpha }_{2}}-\cos \,\theta \frac{{\partial }^{2}}{\partial {\alpha }_{2}\partial \rho })u({\alpha }_{1},{\alpha }_{2},\rho ,d,\theta )=0\\ (\frac{{\partial }^{2}}{\partial {\eta }_{2}\partial {\varepsilon }_{3}}-\frac{{\partial }^{2}}{\partial {\eta }_{3}\partial {\varepsilon }_{2}})u(\varepsilon ;\eta )=-(\frac{\cos \,\theta }{\rho }\frac{\partial L}{\partial {\alpha }_{2}}+\,\sin \,\theta \frac{{\partial }^{2}}{\partial {\alpha }_{2}\partial \rho })u({\alpha }_{1},{\alpha }_{2},\rho ,d,\theta )=0\end{array}$$

Let $\overrightarrow{e}=(\cos \,\theta ,\,\sin \,\theta )$ denote unit vector, therefore ${\overrightarrow{e}}^{\perp }=(-\sin \,\theta ,\,\cos \,\theta )$ is the vector which rotates $\overrightarrow{e}$ by 90° anticlockwise. Furthermore, the operators G and M are used to represent (1/*ρ*)·(*∂L*/*∂d*) + *∂* ^2^/*∂α*_1_ *∂ρ* and (1/*ρ*)·(*∂L*/*∂α*_2_) + *∂* ^2^/*∂α*_2_ *∂ρ*, respectively. Hence, Eq. (15) can be simply written as

16 $$\{\begin{array}{l}Gu\,=\,0\\ {\overrightarrow{e}}^{\perp }Mu\,=\,0\\ \overrightarrow{e}Mu\,=\,0\end{array}$$

Further merge Eq. (16) into Eq. (17)

17 $$\{\begin{array}{l}Gu\,=\,0\\ Mu\,=\,0\end{array}$$

Due to *ρ* and *d* are constants, we would like to express *∂*/*∂r* and *∂*/*∂d* using other variables. Therefore two gradient differential equations are derived firstly, where *l* denotes the line through two points *ε* and *η*

18 $$\begin{array}{rcl}(\eta -\varepsilon )\cdot {\nabla }_{\varepsilon }u(\varepsilon ;\eta ) & = & (\eta -\varepsilon )\cdot {\nabla }_{\varepsilon }{\int }_{R}f(\varepsilon +t(\eta -\varepsilon ))dt\\  & = & {\int }_{R}(\eta -\varepsilon )(1-t)\cdot \nabla f(\varepsilon +t(\eta -\varepsilon ))dt={\int }_{l}(1-t)\frac{df}{dt}dt\end{array}$$

According to integration by parts, Eq. (18) can be expressed as

19 $${\int }_{l}(1-t)\frac{df}{dt}dt=(1-t){f}_{l-\infty }^{l+\infty }-{\int }_{l}-fdt=u(\varepsilon ;\eta )$$

That is,

20 $$(\eta -\varepsilon )\cdot {\nabla }_{\varepsilon }u(\varepsilon ;\eta )=u(\varepsilon ;\eta )$$

Similarly

21 $$(\eta -\varepsilon )\cdot {\nabla }_{\eta }u(\varepsilon ;\eta )=-u(\varepsilon ;\eta )$$

Eqs (20) and (21) can be rewritten with *α*_1_, *α*_2_, *ρ*, *d* and *θ* as follows:

22 $$\begin{array}{rcl}(\eta -\varepsilon )\cdot {\nabla }_{\varepsilon }u(\varepsilon ;\eta ) & = & (\eta -\varepsilon )\cdot (\frac{\partial }{\partial {\varepsilon }_{1}}+\frac{\partial }{\partial {\varepsilon }_{2}};\frac{\partial }{\partial {\varepsilon }_{3}})u(\varepsilon ;\eta )\\  & = & ({\alpha }_{1}{\overrightarrow{e}}^{\perp }-(\rho +d)\overrightarrow{e};{\alpha }_{2})\cdot (\overrightarrow{e}\frac{\partial }{\partial \rho }+\frac{1}{\rho }{\overrightarrow{e}}^{\perp }L;0)u(\varepsilon ;\eta )\\  & = & (\frac{{\alpha }_{1}}{\rho }L-(\rho +d)\frac{\partial }{\partial \rho })u(\varepsilon ;\eta )=u(\varepsilon ;\eta )\end{array}$$

23 $$\begin{array}{rcl}(\eta -\varepsilon )\cdot {\nabla }_{\eta }u(\varepsilon ;\eta ) & = & (\eta -\varepsilon )\cdot (\frac{\partial }{\partial {\eta }_{1}}+\frac{\partial }{\partial {\eta }_{2}};\frac{\partial }{\partial {\eta }_{3}})u(\varepsilon ;\eta )\\  & = & ({\alpha }_{1}{\overrightarrow{e}}^{\perp }-(\rho +d)\overrightarrow{e};{\alpha }_{2})\cdot ({\overrightarrow{e}}^{\perp }\frac{\partial }{\partial {\alpha }_{1}}-\overrightarrow{e}\frac{\partial }{\partial d};\frac{\partial }{\partial {\alpha }_{2}})u(\varepsilon ;\eta )\\  & = & ({\alpha }_{1}\frac{\partial }{\partial {\alpha }_{1}}+{\alpha }_{2}\frac{\partial }{\partial {\alpha }_{2}}+(\rho +d)\frac{\partial }{\partial d})u(\varepsilon ;\eta )=-u(\varepsilon ;\eta )\end{array}$$

After transposition and combining like terms, the expressions of *∂*/*∂ρ* and *∂*/*∂d* are as follows:

24 $$\frac{\partial }{\partial \rho }=\frac{1}{\rho +d}(\frac{{\alpha }_{1}}{\rho }L-1)$$

25 $$\frac{\partial }{\partial d}=\frac{-1}{\rho +d}(1+{\alpha }_{1}\frac{\partial }{\partial {\alpha }_{1}}+{\alpha }_{2}\frac{\partial }{\partial {\alpha }_{2}})$$

With Eqs (24) and (25), Eq. (17) can be expressed as

26 $$\begin{array}{c}0=Gu=(\frac{1}{\rho }\frac{\partial L}{\partial d}+\frac{{\partial }^{2}}{\partial {\alpha }_{1}\partial \rho })u\\ \quad =\frac{1}{\rho +d}(\begin{array}{c}\frac{-{\alpha }_{1}}{\rho (\rho +d)}+(\frac{(\rho -2){\alpha }_{1}^{2}}{\rho (\rho +d)}-\frac{d}{\rho }-1)\frac{\partial }{\partial {\alpha }_{1}}-\frac{2{\alpha }_{1}{\alpha }_{2}}{\rho (\rho +d)}\frac{\partial }{\partial {\alpha }_{2}}-\frac{1}{\rho }\frac{\partial }{\partial \theta }+(\frac{{\alpha }_{1}^{3}(\rho -1)}{\rho (\rho +d)}\\ -\frac{{\alpha }_{1}d}{\rho }+{\alpha }_{1}d)\frac{{\partial }^{2}}{\partial {\alpha }_{1}^{2}}-\frac{{\alpha }_{1}{\alpha }_{2}^{2}}{\rho (\rho +d)}\frac{{\partial }^{2}}{\partial {\alpha }_{2}^{2}}+(\frac{{\alpha }_{1}^{2}{\alpha }_{2}(\rho -2)}{\rho (\rho +d)}-\frac{{\alpha }_{2}d}{\rho })\frac{{\partial }^{2}}{\partial {\alpha }_{1}\partial {\alpha }_{2}}\\ +({\alpha }_{1}-\frac{{\alpha }_{1}}{\rho })\frac{{\partial }^{2}}{\partial {\alpha }_{1}\partial \theta }-\frac{{\alpha }_{2}}{\rho }\frac{{\partial }^{2}}{\partial {\alpha }_{2}\partial \theta }\end{array})u\end{array}$$

27 $$\begin{array}{rcl}0=Mu & = & (\frac{1}{\rho }\frac{\partial L}{\partial {\alpha }_{2}}+\frac{{\partial }^{2}}{\partial {\alpha }_{2}\partial \rho })u\\  & = & (-\frac{1}{\rho +d}\frac{\partial }{\partial {\alpha }_{2}}+(\frac{d}{\rho }+\frac{{\alpha }_{1}d}{\rho (\rho +d)})\frac{{\partial }^{2}}{\partial {\alpha }_{1}\partial {\alpha }_{2}}\\  &  & -(\frac{{\alpha }_{1}}{\rho }+\frac{{\alpha }_{1}^{2}}{\rho (\rho +d)})\frac{{\partial }^{2}}{\partial {\alpha }_{2}\partial d}+\frac{{\alpha }_{1}+\rho +d}{\rho (\rho +d)}\frac{{\partial }^{2}}{\partial {\alpha }_{2}\partial \theta })u\end{array}$$

Finally the equation we have derived is

28 $$\frac{{\partial }^{2}u}{\partial {\alpha }_{2}\partial \theta }=(\frac{\rho }{\rho +d+{\alpha }_{1}}-\frac{{\alpha }_{1}}{\rho +d})\frac{\partial u}{\partial {\alpha }_{2}}-\frac{{\alpha }_{1}{\alpha }_{2}}{\rho +d}\frac{{\partial }^{2}u}{\partial {\alpha }_{2}^{2}}-(\frac{{\alpha }_{1}^{2}}{\rho +d}+d)\frac{{\partial }^{2}u}{\partial {\alpha }_{1}\partial {\alpha }_{2}}$$

The Fourier transform of Eq. (28) is derived with respect to *α*_1_ and *α*_2_. Let u* represent the Fourier transform of *u* and (*k*_1_, *k*_2_) be the corresponding frequency form of (*α*_1_, *α*_2_). Consequently, Eq. (28) is transformed into

29 $$\frac{\partial {u}^{\ast }}{\partial \theta }=\frac{i}{\rho +d}[(\frac{{k}_{1}}{{k}_{2}}-\frac{\rho (\rho +d)}{(\rho +d+{k}_{1}){k}_{2}})\frac{\partial {u}^{\ast }}{\partial {k}_{2}}+(\frac{{k}_{1}^{2}+d(\rho +d)}{{k}_{2}})\frac{{\partial }^{2}{u}^{\ast }}{\partial {k}_{1}\partial {k}_{2}}+{k}_{1}\frac{{\partial }^{2}{u}^{\ast }}{\partial {k}_{2}^{2}}],\,{k}_{2}\ne 0$$

Let *U* denote the right side of (29), in terms of derivative definition, Eq. (29) can be simply expressed as

30 $${u}^{\ast }(\theta +d\theta )={u}^{\ast }(\theta )+d\theta \cdot U,\,{k}_{2}\ne 0$$

Eq. (30) is the JECC equation in the frequency domain. Note that the partial derivatives contained in *U* are consistent with u*(*θ*), e.g., when u*(*θ*) denotes the Fourier transform of the projection at angle *θ*, ∂u*/*∂k*_2_ means ∂u*(*θ*)/*∂k*_2_. To avoid any confusion, we add an angle label to symbol *U*, therefore Eq. (30) can also be written as follows:

31 $${u}^{\ast }(\theta +d\theta )={u}^{\ast }(\theta )+d\theta \cdot U(\theta ),\,\,{k}_{2}\ne 0$$

Or

32 $${u}^{\ast }(\theta )={u}^{\ast }(\theta -d\theta )+d\theta \cdot U(\theta -d\theta ),\,{k}_{2}\ne 0$$

**Figure 2:**
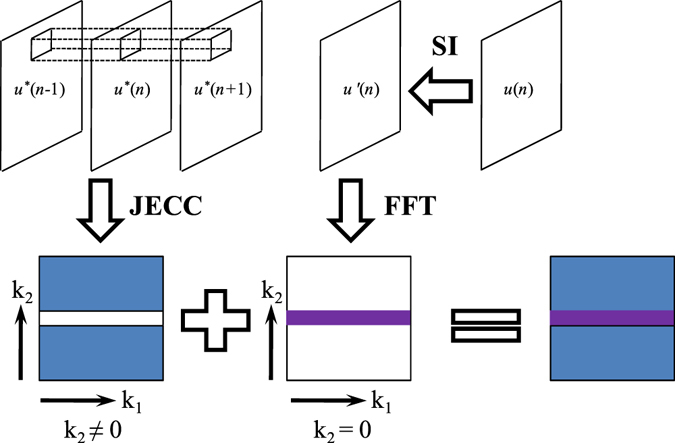
Illustration of the corrupted projection restoration capabilities of JECC and SI in the frequency domain.

As shown in Fig. 2, JECC works well when *k*_2_ ≠ 0 in Eq. (30) and it can restore the area in the blue zone, which represents high-frequency components. However, the zero-frequency component represented by the white horizontal line is beyond the capability of JECC when *k*_2_ = 0 in Eq. (30). To address this issue, a corrupted projection *u* is first interpolated into *u*ʹ via spatial interpolation (SI) in the projection domain, and then *u*ʹ is converted into the frequency domain via fast Fourier transform (FFT) to restore the low-frequency components, which are represented by the purple rectangle. Thus, the proposed method can combine the advantages of JECC and SI to restore corrupted data in the frequency domain. In the following section, we refer to the proposed restoration method as the JECC method.

### Workflow of corrupted projection restoration

**Figure 3:**
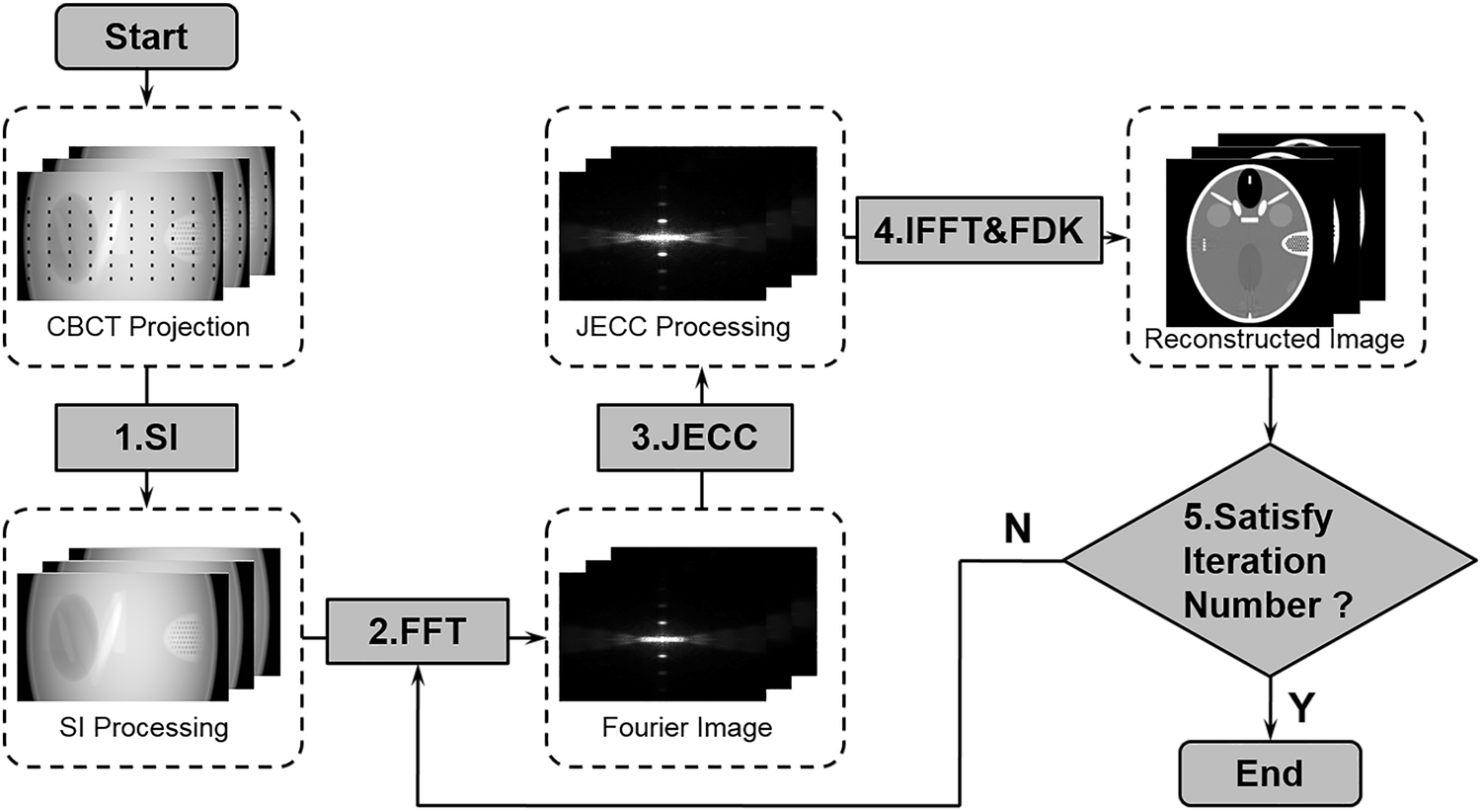
Workflow of the JECC method in circular trajectory CBCT.

Figure 3 shows the flowchart of the JECC method, or the corrupted projection restoration method. The black dots in the projection represent the pixels that should be restored. Let *u*(*θ*) denote the projection at view angle *θ*, and *u*(*θ* + *dθ*) and *u*(*θ* − *dθ*) be the adjacent projections at angle *θ* + *dθ* and *θ* − *dθ*, respectively.

In Step 1, horizontal 1D cubic spline interpolation induces few artifacts because ofthe horizontal shift-invariant weighting in the Feldkamp-Davis-Kress (FDK) algorithm^29^. Thus, cubic spline interpolation is utilized to interpolate the projection in each view to obtain the initial projection denoted by *u* ^I^(*θ*). The purpose of this step is to restore the low-frequency components of the corrupted projection.

In Step 2, *u* ^I^(*θ*), *u*(*θ* + *dθ*), and *u*(*θ* − *dθ*) are converted into frequency domain via Fourier transform. After this step, the corresponding frequency domains *u* ^I*^(*θ*), u*(*θ* + *dθ*), and u*(*θ* − *dθ*) can be obtained.

In Step 3, u*(*θ*) can be restored by substituting u*(*θ* + *dθ*) and u*(*θ* − *dθ*) separately into Eqs (31) and (32). Then, *u* ^R*^(*θ*), which is the updated frequency data at angle *θ*, is a weighted result that combines Eqs (31) and (32) via a factor *ω*. *ω* is a weighting factor which weights the two corresponding results of (31) and (32). The whole derivation is based on the assumption that *u* is differentiable, which is not true in reality. So the method need to combine more restoration information at different direction. In the paper, we set *ω* to 0.5 in order to make two adjacent projections have equal contribution. Subsequently, the initial result *u* ^I*^(*θ*) in Step 2 is updated to *u* ^R*^(*θ*). The JECC equation only holds when *k*_2_ ≠ 0; thus, *u* ^I*^(*θ*) is utilized to extract the low-frequency components when *k*_2_ = 0. In conclusion, this step can be expressed as

33 $${u}^{R\ast }(\theta )=\{\begin{array}{c}\omega ({u}^{\ast }(\theta -d\theta )+d\theta \cdot U(\theta -d\theta ))+(1-\omega )({u}^{\ast }(\theta +d\theta )-d\theta \cdot U(\theta )),\,{k}_{2}\ne 0\\ {u}^{I\ast }(\theta ),\,{k}_{2}=0\end{array}$$

In Step 4, the inverse Fourier transform of *u* ^R*^(*θ*) is utilized to obtain the restoration result by FDK algorithm in the first iteration.

Steps (2)–(4) are repeated until the termination criterion is satisfied. In this study, we set the iteration number as the termination criterion because the restored value remains stable after a certain number of iterations.

## Experiments

### Moving BSA scatter correction case

**Figure 4:**
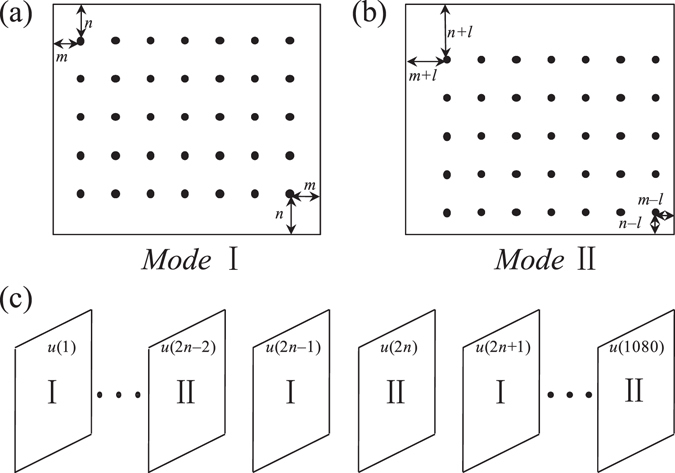
Configuration of moving BSA scatter correction case. (**a**) BSA in odd-numbered views (mode I); (**b**) BSA in even-numbered views (mode II); (**c**) projections in a circular trajectory by moving the BSA scatter correction protocol. The odd-numbered views use mode I in (**a**), and the even-numbered views use mode II in (**b**).

In this section, the JECC method is applied to the simulation of a BSA scatter correction case. As shown in Fig. 4(a) and (b), the BSA dimension is 15 × 7 with each blocker shading 5 × 5 pixels, which are denoted by black dots. In this study, BSA is fixed at the same position in odd-numbered views, as shown in Fig. 4(a), whereas it moves to another location in even-numbered views, as shown in Fig. 4(b). In practice, we divide the projections into two categories: the odd-numbered views use mode I and the even-numbered views use mode II as shown in Fig. 4(c). The movement distance *l* of BSA between the odd-numbered and even-numbered views is 7 pixels. The phantom we tested is a head phantom called FORBILD, which has rich high-frequency details. The dimension of the flat panel detector is 850 × 200, and its cell size is 1 × 1 mm^2^. Both *ρ* and *d* are 500 mm. The number of projections is 1080. Each iteration result is compared with the result of the SI method.

### Abnormal pixel correction case

As a result of a deficient semiconductor in the flat panel detector or a damage caused by improper operation, defective cells in the detector become inconsistent with the responses of the X-ray photons. The presence of defective cells among the hundreds of thousands of cells in the detector is normal. These defective cells cause abnormal pixels in raw projections. However, these abnormal pixels can be detected in a projection by repeatedly scanning the projection at various exposure levels. In general, the detector manufacturer provides the abnormal pixel detection and correction protocol. In such protocol, the pixel value that corresponds to the defective cell is linearly interpolated using nearby pixels. However, linear interpolation (LI) typically causes streak artifacts, and artifacts caused by abnormal pixels in one of hundreds of 2D slices are difficult to find among hundreds of 2D slices. The abnormal pixel correction protocol is related to corrupted projection restoration; thus, the JECC method is also utilized in this case.

**Figure 5:**
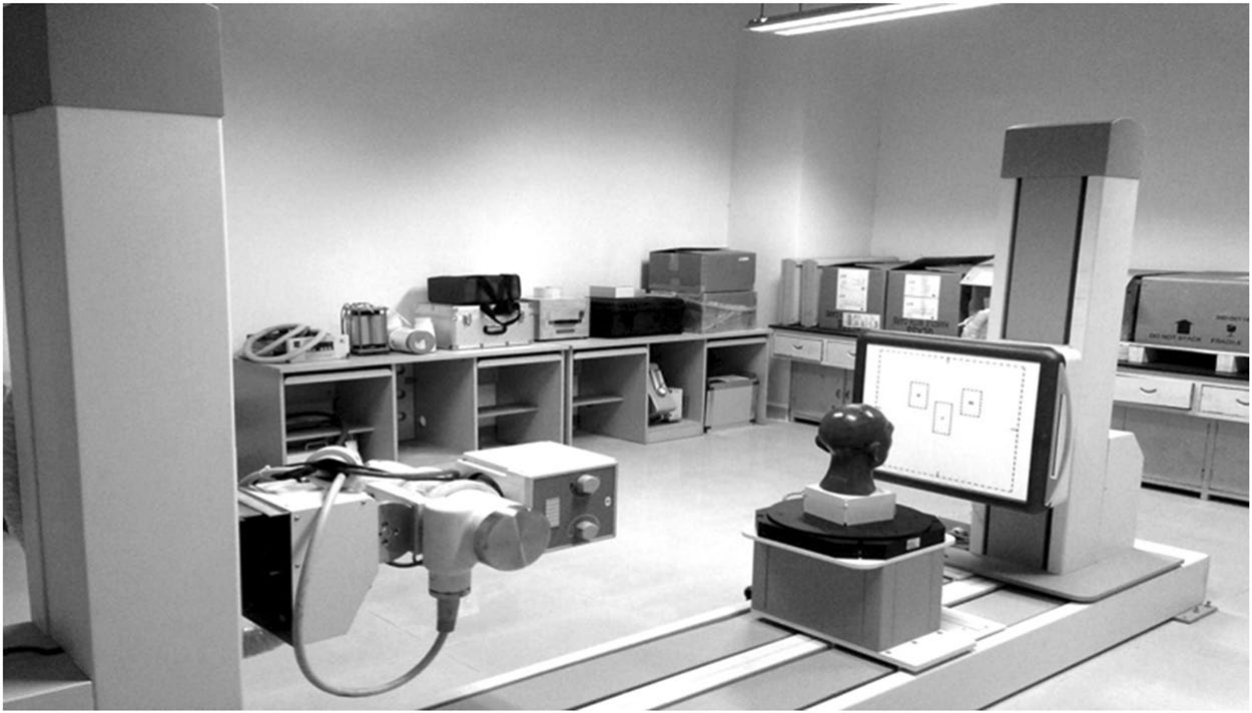
In-house bench-top CBCT system for raw projection acquisition.

As shown in Fig. 5, we acquired the raw projections using an in-house bench-top CBCT system, in which the CBCT scanning protocol is presented in Table 2. We then simulated the abnormal pixels induced by the defective detector cells, as illustrated in detail in a previous paper^1^. Furthermore, to further verify the capability of the proposed method, we have tested it in a more challenging case with real data acquired by a detector which has a group of corrupted cells.

**Table 2:** System setting for circular trajectory CBCT.

Source-to-axis distance, *ρ*	100 cm
Axis-to-detector distance, *d*	50 cm
Detector dimension	1024 × 768
Detector size	40 × 30 cm^2^
Number of projections over 360°	360
Phantom dimension	512 × 512 × 100
Voxel size	0.037 × 0.037 × 0.0625 cm^3^

### Metal artifact reduction case

In terms of CBCT in the circular trajectory geometry, raw 2D projections are firstly acquired by rotating over 360°. Then the transection images are reconstructed by FDK algorithm. For segmenting the metal objects from human tissue, the global threshold method is used in each 2D reconstructed image. In this workflow, all voxels above 3000 HU will be considered as metal voxels. After metal segmentation by threshold, the metal regions are recognized. Subsequently, SI method or the proposed method is selected to restore the corrupted data of the acquired projections with the guidance of metal-only measurement data generated by forward projecting metal images. Then, the corrected image can be reconstructed with the corrected projection data. After the reconstruction, the segmented metal objects are finally reinserted to display the implants. In this case, the projection correlation based view interpolation (PC-VI) method proposed by Yan^16^ is also employed to compare the capability of the JECC method.

### Performance evaluation

#### Evaluation by error comparison

For quantitative measurement, we selected the mean absolute error (MAE) to measure the difference between the conventional SI method and the proposed method. MAE is defined as

34 $$MAE=\frac{{\sum }_{i=1}^{N}|\mu (i)-{\mu }_{t}(i)|}{N}$$

where *μ*(*i*) denotes the *i*th pixel value in the reconstructed image, *μ*_*t*_(*i*) represents the *i*th pixel value in the reference image, and *N* is the number of total pixels.

#### Evaluation by noise reduction

For quantitative comparison, we also used signal-to-noise ratio (SNR) to evaluate noise reduction in the images. SNR is defined as

35 $$SNR=10{\mathrm{log}}_{10}(\frac{{\sum }_{i}^{N}{(\mu (i)-{\mu }_{m})}^{2}}{{\sum }_{i}^{N}{(\mu (i)-{\mu }_{t}(i))}^{2}})$$

where *μ*_*m*_ denotes the mean value of the reconstructed image. The definitions of the other symbols are the same as those in Eq. (34).

#### Evaluation by image similarity

In this study, the universal quality index (UQI)^30^ was utilized to assess the degree of similarity at the region of interest (ROI) in detail. When the ROI is given, the associative mean, variance, and covariance of the ROI can be defined as:

36 $$\bar{\mu }=\frac{{\sum }_{i=1}^{M}\mu (i)}{M}$$

37 $${\sigma }^{2}=\frac{{\sum }_{i=1}^{M}{(\mu (i)-\bar{\mu })}^{2}}{M-1}$$

38 $$Cov\{\mu ,{\mu }_{t}\}=\frac{{\sum }_{i=1}^{M}((\mu (i)-\bar{\mu })({\mu }_{t}(i)-{\bar{\mu }}_{t}))}{M-1}$$

where *M* represents the number of pixels within the ROI. Then, UQI is defined as

39 $$UQI=\frac{4Cov\{\mu ,{\mu }_{t}\}}{{\sigma }^{2}+{\sigma }_{t}^{2}}\cdot \frac{\bar{\mu }\cdot {\bar{\mu }}_{t}}{{\bar{\mu }}^{2}+{\bar{\mu }}_{t}^{2}}$$

UQI shows the similarity level between the two images with a value ranging from 0 to 1. A UQI value closer to 1 indicates that the similarity is high between the reconstructed and reference images.

## Results

### Moving BSA scatter correction results

#### Visualization-based evaluation

**Figure 6:**
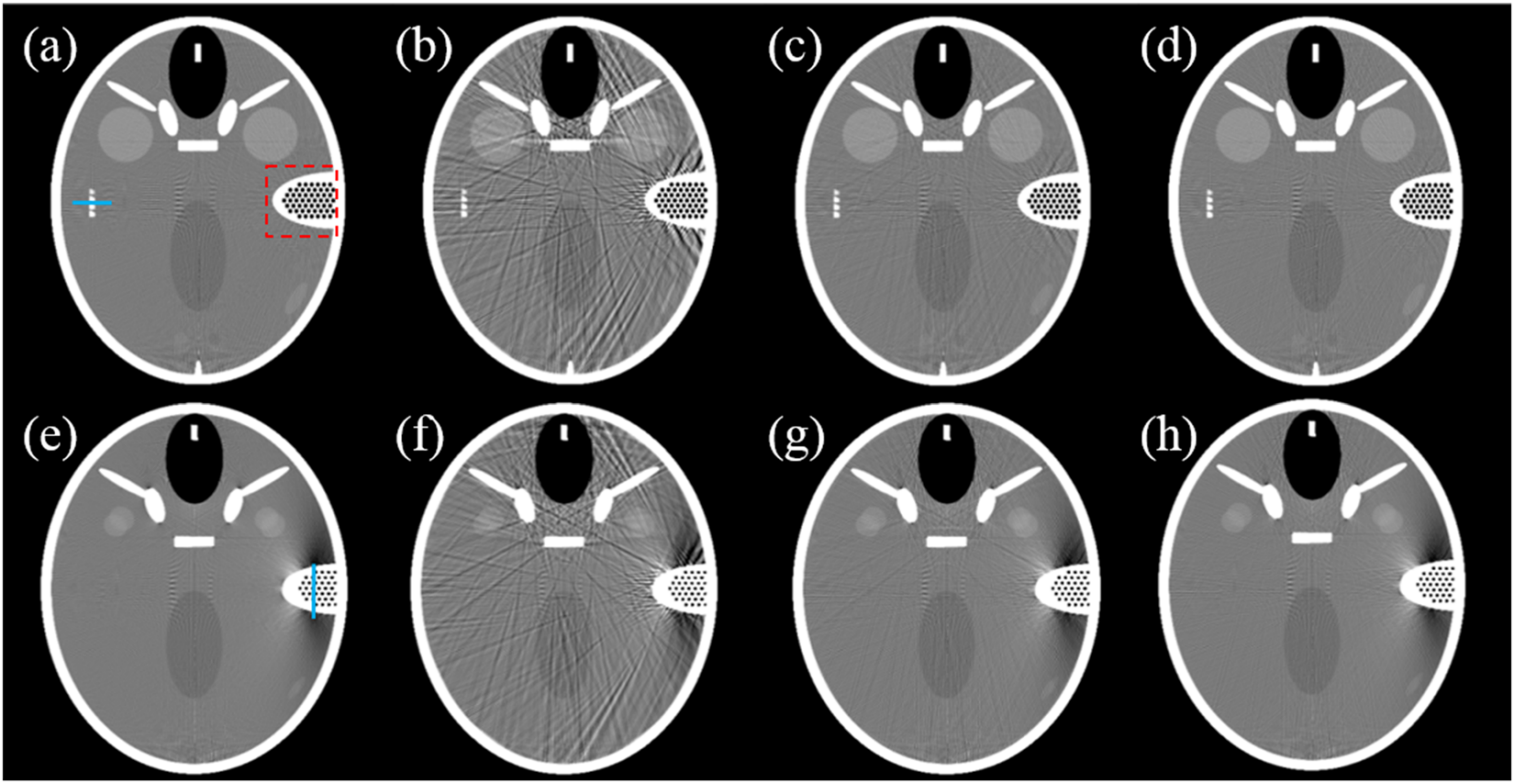
JECC performance on different slices. (**a**) Reference image on the 100th slice; (**b**–**d**) images on the 100th slice reconstructed using the SI method, JECC 1st iteration, and JECC 4th iteration, respectively; (**e**) reference image on the 80th slice; (**f–h**) images on the 80th slice reconstructed using the SI method, JECC 1st iteration, and JECC 4th iteration, respectively.

The experimental results were compared and analyzed through visual inspection. Figure 6(a–d) show the reference image and the images on the 100th slice reconstructed using the SI method, JECC 1st iteration, and JECC 4th iteration, respectively. Figure 6(e–h) show the reference image and the images on the 80th slice reconstructed using the SI method, JECC 1st iteration, and JECC 4th iteration, respectively. The images reconstructed using the JECC method are visually better than those reconstructed using the SI method. Compared with the SI method, the JECC method achieves good performance in cases of LI-induced streak artifacts. We can obtain a visually satisfactory image quality after the JECC 4th iteration.

#### Profile-based evaluation

**Figure 7:**
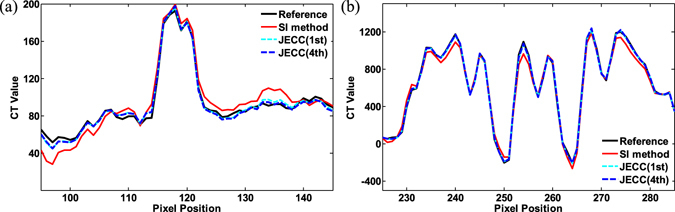
Image profiles of the results in Fig. 6. (**a**) Profiles across the 95th to 145th columns in the 255th row of the results indicated by the blue line in Fig. 6(a); (**b**) profiles across the 225th to 285th rows in the 401th column of the results indicated by the blue line in Fig. 6(e).

Figure 7(a and b) plot the vertical profiles on the 80th slice and the 100th slice, respectively. The profiles of the results of the JECC method are considerably closer to those of the reference image than those of the results of the conventional SI method. The SI method causes more fluctuations around the reference value, and this result is consistent with the appearance of streak artifacts in the image. The results also indicate that the proposed method can achieve better profiles compared with the SI method.

#### Quantitative evaluation

The difference between the reference image and the images reconstructed using the conventional SI method and the JECC method are quantitatively evaluated by measuring the MAEs of the reconstructed results in different views. The calculated MAEs with the projection views of 135, 270, 360, 540, and 1080 are shown in Table 3. Compared with the SI method, the first iteration of the JECC method results in an appreciable improvement. Moreover, as the iteration number increases, the MAEs decrease. At the fourth iteration, the JECC method achieves the lowest MAE. After four iterations, the proposed method outperforms the traditional correction method (i.e., SI), demonstrating 72.21% reduction in terms of MAE. The MAEs have not been significantly changed by the fourth iteration onward.

**Table 3:** MAE comparison in different views.

MAE (10^−4^)	SI	JECC (1st)	JECC (2nd)	JECC (3rd)	JECC (4th)
135 views	7.9679	2.6999	2.3268	2.0605	1.9965
270 views	5.6604	2.003	1.7853	1.5939	1.5027
360 views	4.5826	1.9754	1.5426	1.2486	1.2136
540 views	3.9839	1.4605	1.2930	1.1573	1.1432
1080 views	1.9905	0.7307	0.6469	0.5791	0.5532

Furthermore, SNR is used to quantitatively evaluate the noise reduction of the present method. The results are presented in Table 4. From 135 views to the 1080 views, the SNR gains of the JECC method compared with that of the SI method are between 7 dB and 3.6 dB. Compared with the SI method, the present method yields higher SNR. This result demonstrates that the present method can achieve noticeable gains in terms of noise and artifact suppression.

**Table 4:** SNR measurements using projections in 135 views to 1080 views.

SNR (dB)	SI	JECC (1st)	JECC (2nd)	JECC (3rd)	JECC (4th)
135 views	13.6522	18.8312	20.3761	20.5832	20.6032
270 views	16.6613	19.7832	21.8766	22.0738	22.1293
360 views	19.7218	22.3037	23.6489	23.8731	23.8812
540 views	21.7632	23.5316	24.9843	25.0983	25.1380
1080 views	22.8618	24.0872	25.0012	25.2237	25.2342

**Figure 8:**
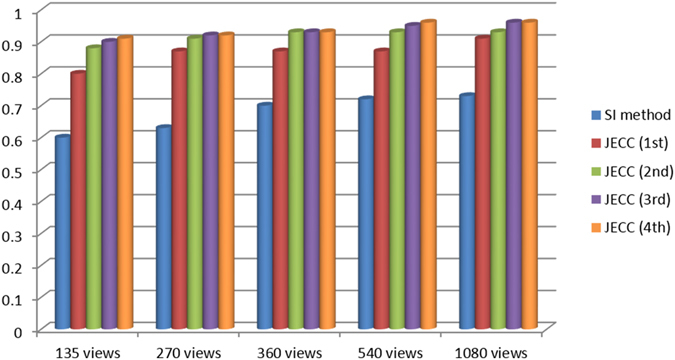
Comparison of the different methods in terms of UQI.

To evaluate image similarity between the reconstructed results and the reference image, we selected the ROI marked with a red dashed square in Fig. 6(a) to calculate the UQI scores. The corresponding UQI scores are shown in Fig. 8. In all the views, the present method yields higher UQI scores (over 0.9), thereby implying that it outperforms the traditional method in terms of UQI by over 0.24 on average.

### Abnormal pixel correction results

#### Visualization-based evaluation

**Figure 9:**
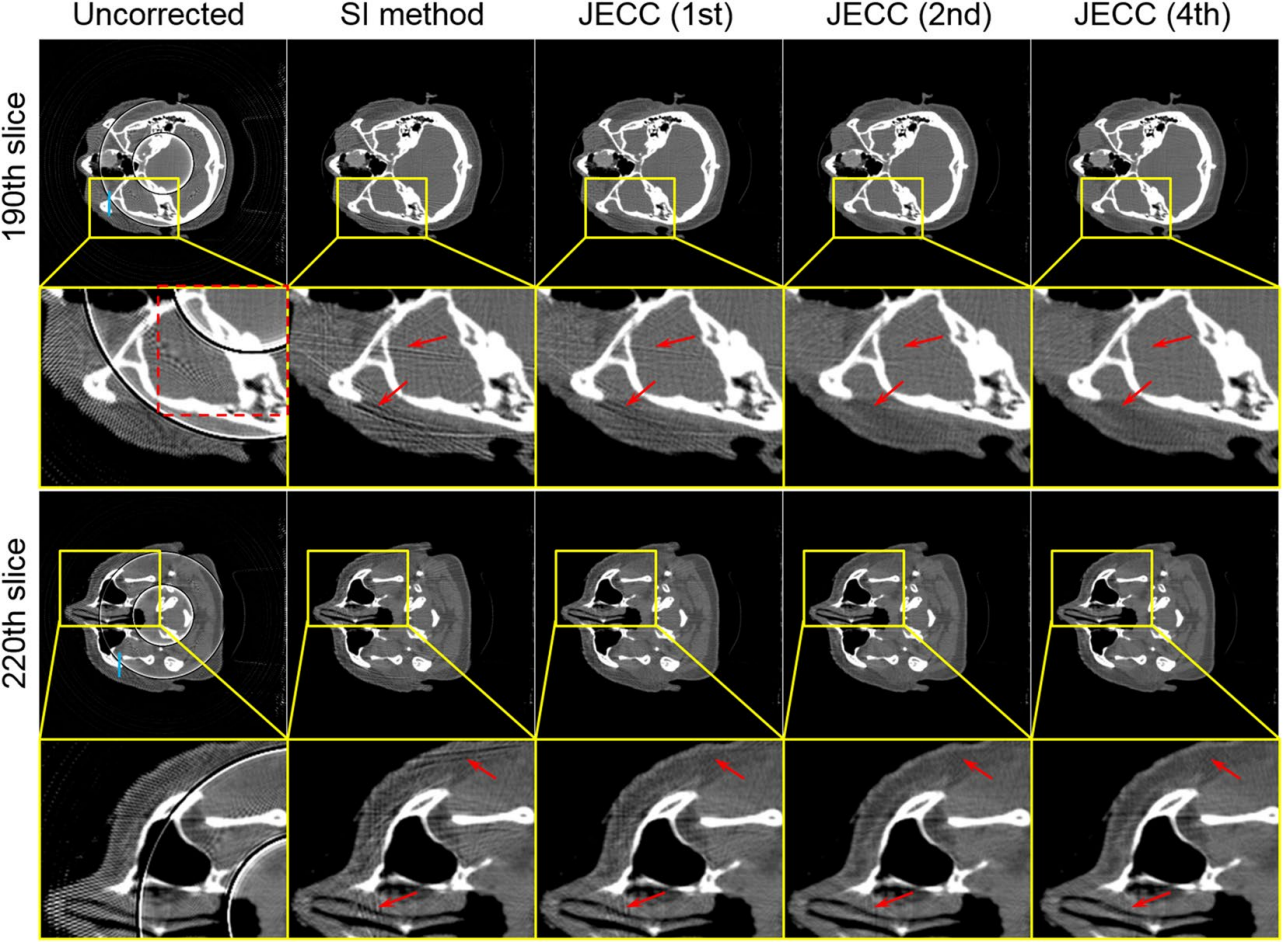
Image without correction and images reconstructed using the SI method and the JECC method. The first row presents the images on the 190th slice, and the third row presents the images on the 220th. The second and fourth rows present the magnified ROIs marked with yellow squares in the images in the first and third rows.

Figure 9 shows the reconstructed images without correction, corrected with SI method and the JECC method on the 190th slice and 220th slice. As shown in the first column of Fig. 9, the abnormal pixels induce ring artifacts. As shown in the second column, the conventional SI method can efficiently remove the ring artifacts. A satisfactory image quality can be acquired using the JECC method after four iterations. The zoomed ROIs in Fig. 9 show that several streak artifacts, which are indicated by the red arrows, are still present. These streak artifacts degrade image quality severely. However, such artifacts are mitigated in the images in the third and fourth columns, and these artifacts even disappear visually in the images in the fifth column, which shows the results obtained after four iterations of the JECC method.

**Figure 10:**
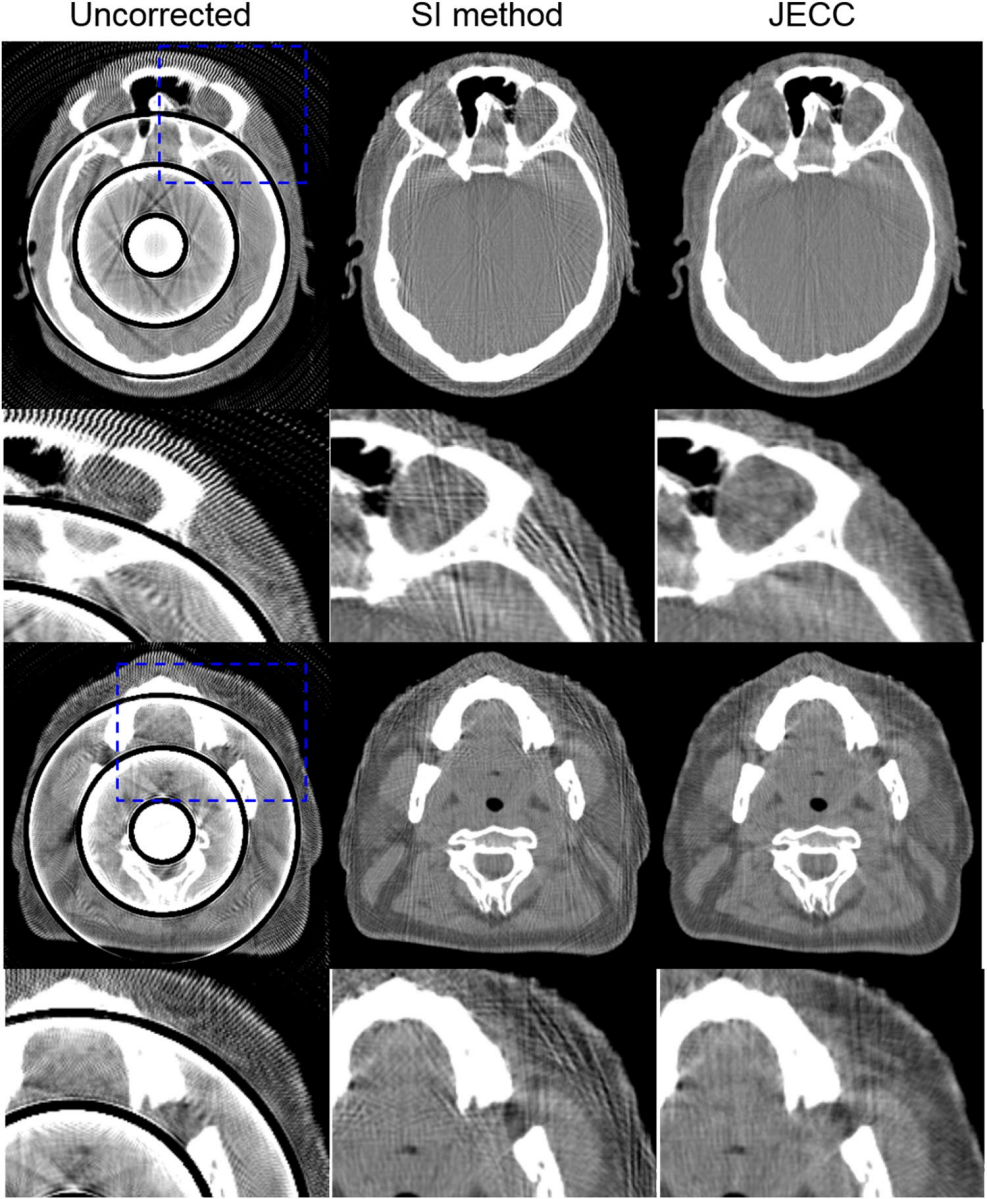
Images corrected with SI method and the proposed method. The first and third rows present two representative slices. The second and fourth rows present the zoomed ROIs marked with blue dashed squares in the first and third rows.

Figure 10 displays one more challenging case with ring artifacts. As shown in the first column, uncorrected images suffer from severe bending artifacts caused by a group of blocked pixels on detector. The ring artifacts are so wide that they degrade image diagnosis severely. The second column represents the results of SI method. SI method can reduce dark ring artifacts at the cost of newly introduced streak artifacts. SI method estimates the missing data by utilizing nearby uncorrupted data, therefore the interpolated data deviates from original true values in the dark shadow. When the corrupted regions are large, the severe deviations will unavoidably lead to new streak artifacts. However, due to the complete utilization of the information of adjacent projections, the proposed method outperforms SI method in both artifact suppression and bone structure preservation, which can be observed in the zoomed ROIs in the Fig. 10.

#### Profile-based evaluation

**Figure 11:**
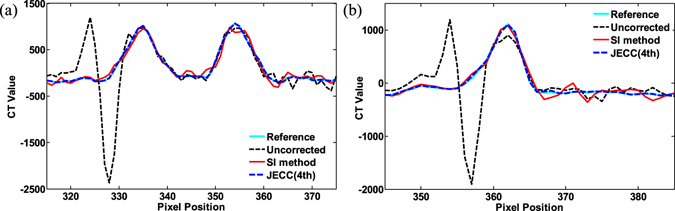
Image profiles of the results in Fig. 9. (**a**) Profiles across the 315th to 375th rows in the 145th column of the results indicated by a blue line in the top row in Fig. 9; (**b**) profiles across the 345th to 385th rows in the 170th column of the results indicated by a blue line in the third row in Fig. 9.

Figure 11(a and b) plot the horizontal profiles on the 190th slice and 220th slice. The profiles of the results of the JECC method are considerably closer to those of the reference image than those of the results of the SI method. The profile of the image without any correction does not match the reference well because of the ring artifacts; this result partially proves that ring artifacts seriously degrade the quality of images. The use of the SI method allows for the improvement of image quality; however, the fluctuation in the profile demands for further improvement. By contrast, the JECC method achieves high image quality, as depicted by the blue dashed line in Fig. 11(a and b).

#### Quantitative evaluation

Table 5 shows the MAEs of the results reconstructed using the different algorithms in various views. With respect to the uncorrected image results, the MAEs of the results obtained using the JECC method is reduced by 9.06, 8.23, 7.77, 7.65, and 7.38 in the 135 views, 270 views, 360 views, 540 views and 1080 views cases, respectively. With respect to the conventional SI method results, the MAEs of the results obtained using the JECC method is reduced by 5.16, 8.23, 3.98, 2.71, and 2.86 in the 135 views, 270 views, 360 views, 540 views and 1080 views cases, respectively.

**Table 5:** MAE comparison in different views.

MAE (10^−4^)	Uncorrected	SI method	JECC (1st)	JECC (2nd)	JECC (3rd)	JECC (4th)
135 views	11.2322	7.3233	3.8762	2.4329	2.1721	2.1673
270 views	10.1293	5.8788	2.6731	2.2182	2.0865	1.9031
360 views	9.2321	4.8204	2.3518	1.8932	1.4622	1.4653
540 views	9.0211	4.0876	1.8736	1.5627	1.3728	1.3727
1080 views	8.2833	3.7540	1.0821	0.9876	0.8937	0.8988

In addition, the SNRs of the results are presented in Table 6. Compared with the SI method and the uncorrected results, the present method yields relatively higher SNRs. From 135 views to 1080 views, the SNRs of the JECC method increased by 5.44 dB to 1.99 dB compared with the conventional SI method. Thus, the present method can achieve noticeable SNR gains over the conventional SI method.

**Table 6:** SNR measurements from 135 views to 1080 views cases.

SNR (dB)	Uncorrected	SI method	JECC (1st)	JECC (2nd)	JECC (3rd)	JECC (4th)
135 views	12.3757	15.1356	18.4523	20.2832	20.5723	20.5769
270 views	13.6894	18.4674	20.3865	20.9837	21.0932	21.1921
360 views	15.8732	20.2340	23.6748	24.0983	24.4895	24.4896
540 views	18.9871	22.4067	24.8764	24.9873	25.1021	25.1073
1080 views	19.0821	23.2357	24.9872	25.0832	25.2184	25.2294

**Figure 12:**
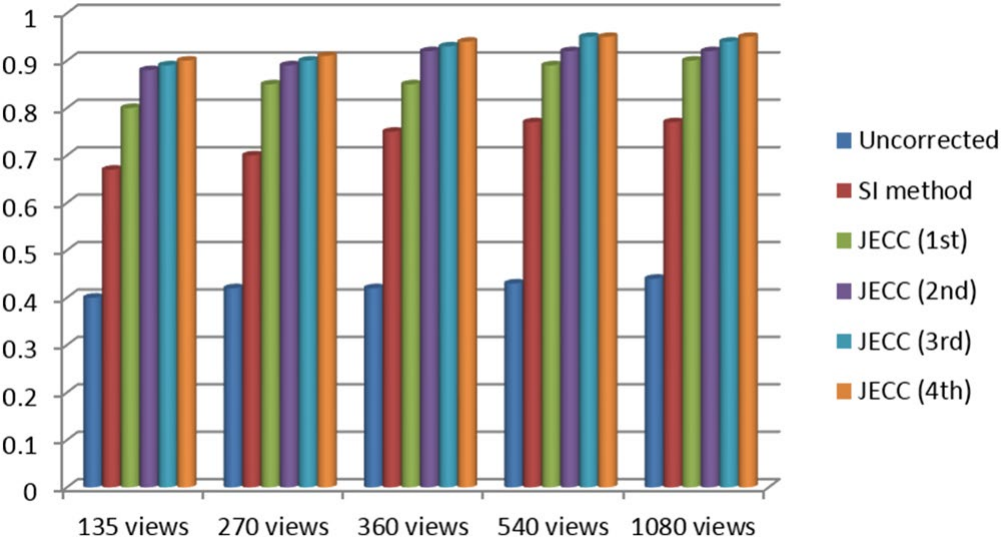
Comparison of the different methods in terms of UQI.

In this case, we selected the ROI marked with a red dashed square in Fig. 9 to calculate the UQI scores. The corresponding UQI scores are shown in Fig. 12. In all the cases, the present method yields UQI scores that are 0.51 higher than those of the uncorrected image and 0.19 higher than those of the SI method. These results partially demonstrate that the JECC method is a better solution for restoring local detailed information.

### Metal artifact reduction results

#### Visualization-based evaluation

**Figure 13:**
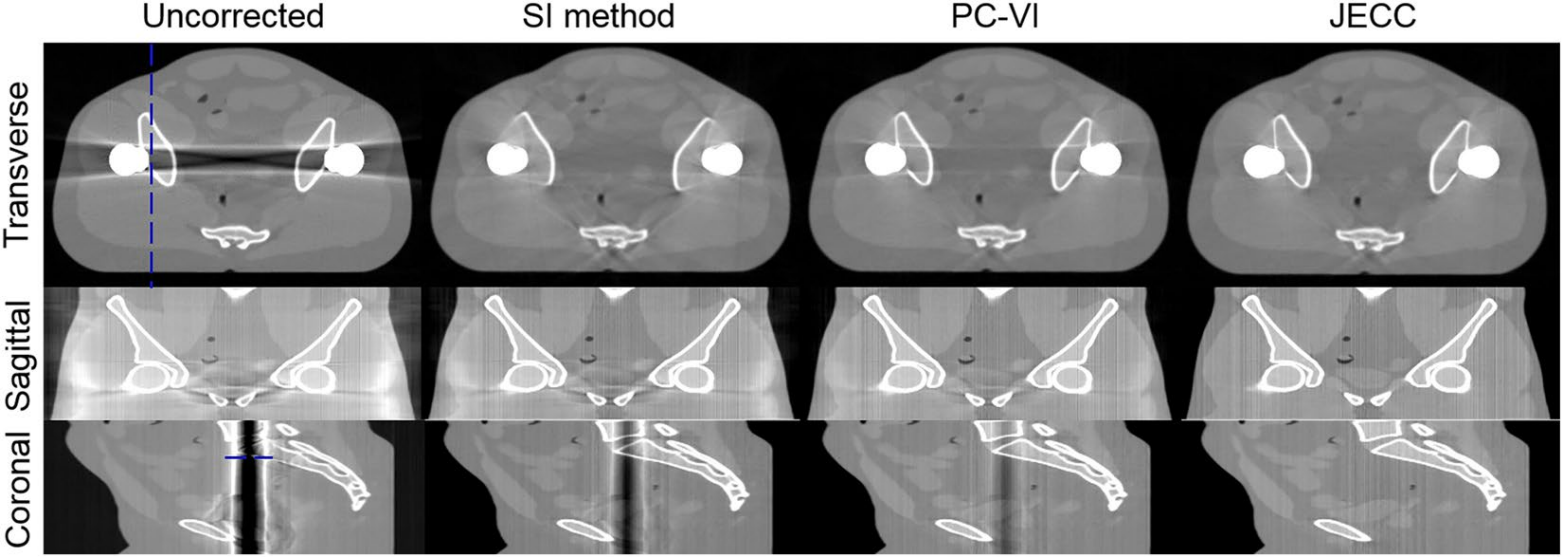
Bilateral hip prostheses simulation. The transverse, sagittal, and coronal views are displayed in the first, second, and third rows, respectively.

To evaluate the capability of JECC method, one anthropomorphic male pelvic phantom (CIRS Inc., Norfolk, VA, USA) was used to simulate bilateral hip prostheses. The reconstructed images without correction, corrected with SI method, PC-VI, and JECC method are shown in Fig. 13, respectively. Transverse and coronal views indicate that the uncorrected image suffers from severe beam hardening artifacts between bilateral metal prostheses. In sagittal view, there are severe streak artifacts reducing image quality. All SI method, PC-VI, and JECC method can reduce those metal artifacts to varying degrees. However, SI method only removes some streak artifacts at the cost of the loss of bone structures surrounding the metal prostheses. As for PC-VI, though the bone structures lost in the result of SI method are preserved, the existence of residual streak artifacts observed in the transverse and coronal views contaminates the tissues between bone structures as seen from the sagittal view. Compared with above two methods, JECC method achieves better performance in both the preservation of bone edge structures and the suppression of metal artifacts in the transverse, sagittal, or coronal views.

**Figure 14:**
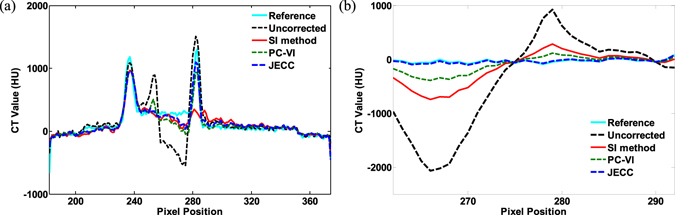
Image Profiles of the results in Fig. 13. (**a**) Profiles indicated by the blue dashed line in transverse view; (**b**) profiles indicated by the blue dashed line in coronal view.

Figure 14(a and b) show the profiles indicated by the blue dashed line in transverse and coronal views, respectively. Figure 14 indicates that the profile line of JECC is closer to that of the reference than those of SI and PC-VI. More fluctuations around the reference value imply the loss of fine bone edge structures and the appearance of metal artifacts. These profiles also demonstrate that JECC can gain better results compared with other two methods.

**Figure 15:**
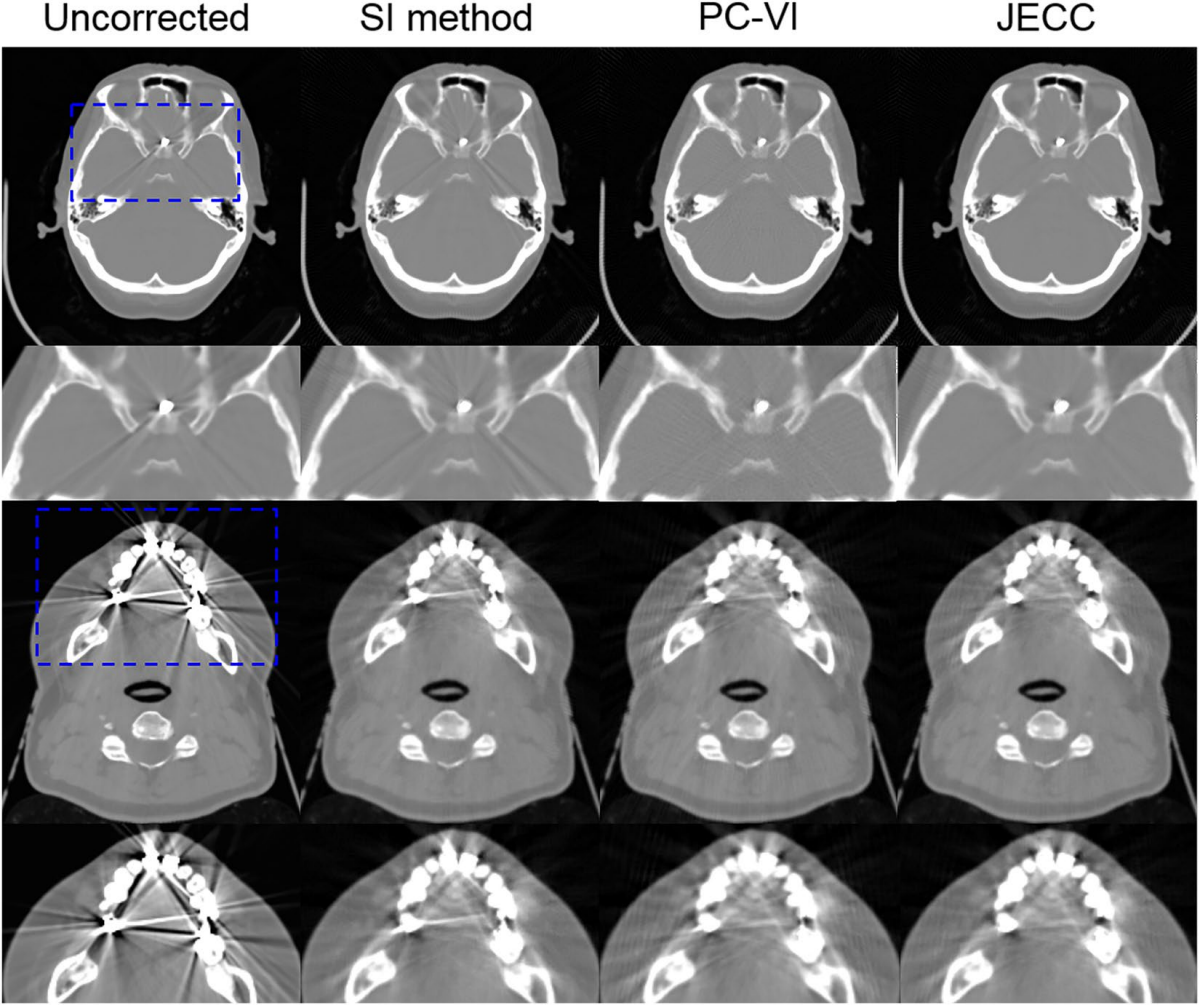
Two clinical cases are displayed here. The first row is the head case with a brain stimulator and the second row presents the corresponding magnified ROIs which are the blue dashed squares containing the metal implant, the bottom two rows present the dental case with three dental fillings on the back teeth and one dental filling on the front tooth.

Furthermore, the performance of JECC method was further demonstrated by clinical measurement data. As shown in Fig. 15, two different kinds of clinical cases were tested in this work. The first row is a head case with a brain stimulator, and the third row is a case with multiple dental fillings which is referred to as the most challenging in the field of metal artifact reduction due to dental dense characteristic and irregular edge shape. In the head case, it is obviously that these radial streak artifacts tangent to metal stimulator severely degrade the uncorrected image quality. The correction result of SI is even worse than the uncorrected image due to failure to reduce original artifacts and some newly introduced artifacts. However, PC-VI and JECC method can get visually satisfactory image quality as seen from zoomed ROIs in the second row in Fig. 15. The correction results for dental case which is classified as the worst situation are shown in the bottom two rows. The beam hardening artifacts between four dental fillings and severe streak artifacts protruding from the corner of each filling are significantly present in the uncorrected image. SI method removes dark beam hardening artifacts considerably but the tooth edges are blurred due to the inaccurate information restored by interpolation. Some bright artifacts in the uncorrected image disappear after the correction with PC-VI, but some streak artifacts still surround dental fillings. However, there are mildest artifacts remaining in the result of JECC method. Furthermore, the dental edges blurred by SI method are preserved significantly. All in all, JECC method can render an overall improvement of image quality.

## Discussion

In this study, we derive a new consistency condition from John’s equation for the circular trajectory CBCT. As shown in the scatter correction, abnormal pixel correction, and metal artifact reduction experiments, the proposed restoration method can obtain a visually satisfactory image quality. The quantitative results of the present method are significantly better than those of the conventional method for different slices and view numbers. This outcome can be ascribed to the complete utilization by the present method of the information of adjacent projections. By contrast, the conventional SI method only utilizes the information within the current projection to restore corrupted data in each projection.

**Figure 16:**
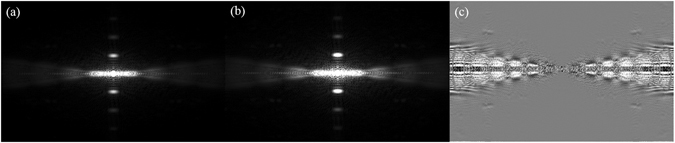
Frequency domain illustrations of the same image. (**a**) Frequency domain of the image obtained using the SI method; (**b**) frequency domain of the image obtained using the JECC method; (**c**) frequency domain of the difference between the images in (**a**) and (**b**).

In addition, we wish to discuss other relevant issues. As shown in Eq. (30), the adjacent projections, i.e., u*(*θ* + *dθ*) and u*(*θ* − *dθ*), can be separately substituted into JECC to calculate the restored projection u*(*θ*). To explore the Fourier properties of JECC, we compare the changes before and after utilizing JECC within the same image in the frequency domain. Figure 16(a and b) show the frequency domain of the image with the SI method and the proposed method, respectively. Figure 16(c) presents the difference by subtracting Fig. 16(a) from 16(b). Figure 16(c) shows that JECC can restore more frequency information of the corrupted projections, particularly their high-frequency components.

Another crucial point we should pay attention to is that the John’s equation related deviation is based on the assumption that the image *u* is reasonably differentiable. In fact, this is inaccurate, e.g., due to the sharp jump at boundary of tissues. However, in view of the complete utilization of the information of adjacent projections, this drawback of John’s equation relevant consistency condition can be ignored.

## Conclusion

In this study, a new consistency condition from John’s equation is derived to restore corrupted projections in circular trajectory CBCT. The proposed method is tested in moving BSA scatter correction, metal artifact reduction, and abnormal pixel correction. The performance results verify that the proposed JECC method is useful and effective in restoring lost data of corrupted projections in circular trajectory CBCT.

## References

[1] Tang X, Ning R, Yu R, Conover D (2001). Cone beam volume CT image artifacts caused by defective cells in x-ray flat panel imagers and the artifact removal using a wavelet-analysis-based algorithm. Med. Phys..

[2] Prell D, Kyriakou Y, Kalender WA (2009). Comparison of ring artifact correction methods for flat-detector CT. Phys. Med. Biol..

[3] Anas EMA, Kim JG, Lee SY, Hasan MK (2011). Comparison of ring artifact removal methods using flat panel detector based CT images. Biomed. Eng. Online..

[4] Anas EMA, Lee SY, Hasan MK (2011). Classification of ring artifacts for their effective removal using type adaptive correction schemes. Comput. Biol. Med..

[5] Anas EMA, Lee SY, Hasan MK (2010). Removal of ring artifacts in CT imaging through detection and correction of stripes in the sinogram. Phys. Med. Biol..

[6] Ning R, Tang X, Conover D (2004). X-ray scatter correction algorithm for cone beam CT imaging. Med. Phys..

[7] Zhu L, Xie Y, Wang J, Xing L (2009). Scatter correction for cone-beam CT in radiation therapy. Med. Phys..

[8] Shi L (2016). Corrigendum: Improving Low-dose Cardiac CT Images based on 3D Sparse Representation. Sci. Rep..

[9] Floyd CE, Chotas HG, Ravin CE (1994). Scatter-reduction characteristics of an infinity-focused gridded radiographic cassette. Invest. Radiol..

[10] Graham S, Moseley D, Siewerdsen J, Jaffray D (2007). Compensators for dose and scatter management in cone-beam computed tomography. Med. Phys..

[11] Poludniowski G, Evans P, Hansen V, Webb S (2009). An efficient Monte Carlo-based algorithm for scatter correction in keV cone-beam CT. Phys. Med. Biol..

[12] Xu Y (2015). A practical cone-beam CT scatter correction method with optimized Monte Carlo simulations for image-guided radiation therapy. Phys. Med. Biol..

[13] Yao W, Leszczynski KW (2009). An analytical approach to estimating the first order x-ray scatter in heterogeneous medium. Med. Phys..

[14] Meyer M, Kalender WA, Kyriakou Y (2010). A fast and pragmatic approach for scatter correction in flat-detector CT using elliptic modeling and iterative optimization. Phys. Med. Biol..

[15] Cai W, Ning R, Conover D (2008). Simplified method of scatter correction using a beam-stop-array algorithm for cone-beam computed tomography breast imaging. Opt. Eng..

[16] Yan H, Mou X, Tang S, Xu Q, Zankl M (2010). Projection correlation based view interpolation for cone beam CT: primary fluence restoration in scatter measurement with a moving beam stop array. Phys. Med. Biol..

[17] Love LA, Kruger RA (1987). Scatter estimation for a digital radiographic system using convolution filtering. Med. Phys..

[18] Zhu L, Strobel N, Fahrig R (2005). X-ray scatter correction for cone-beam CT using moving blocker array. Med. Img..

[19] Meyer E (2010). Normalized metal artifact reduction (NMAR) in computed tomography. Med. Phys..

[20] Wang J (2013). Metal artifact reduction in CT using fusion based prior image. Med. Phys.

[21] Meilinger M (2011). Metal artifact reduction in cone beam computed tomography using forward projected reconstruction information. Z. Med. Phys..

[22] Clackdoyle R, Desbat L (2013). Full data consistency conditions for cone-beam projections with sources on a plane. Phys. Med. Biol..

[23] Chen G-H, Leng S (2005). A new data consistency condition for fan-beam projection data. Med. Phys..

[24] John F (1938). The ultrahyperbolic differential equation with four independent variables. Duke Math J..

[25] Tang S, Xu Q, Mou X, Tang X (2012). The mathematical equivalence of consistency conditions in the divergent-beam computed tomography. J. X-ray Sci. Technol..

[26] Sidky EY, Zou Y, Xia D, Pan X (2005). A consistency condition for cone-beam CT with general source trajectories. Med. Img..

[27] Patch SK (2002). Computation of unmeasured third-generation VCT views from measured views. IEEE Trans. Med. Img..

[28] Patch SK (2002). Consistency conditions upon 3D CT data and the wave equation. Phys. Med. Biol..

[29] Feldkamp LA, Davis LC, Kress JW (1984). Practical cone-beam algorithm. J. Opt. Soc. Am. A.

[30] Wang Z, Bovik AC (2002). A universal image quality index. IEEE Signal Process. Lett..

